# The Adverse Impact of Bisphenol A Exposure on Optimal Cardiovascular Health as Measured by Life’s Essential 8 in U.S. Adults: Evidence from NHANES 2005 to 2016

**DOI:** 10.3390/nu16193253

**Published:** 2024-09-26

**Authors:** Yemei Chen, Chao Xu, Ying Huang, Zhaoyan Liu, Jiupeng Zou, Huilian Zhu

**Affiliations:** 1 Department of Nutrition, School of Public Health, Sun Yat-Sen University, 74 Zhong Shan Road 2, Guangzhou 510080, China; chenym263@mail3.sysu.edu.cn (Y.C.); liuzhy235@mail.sysu.edu.cn (Z.L.); zoujp7@mail2.sysu.edu.cn (J.Z.); 2 Department of Clinical Nutrition, The First Affiliated Hospital of Guangzhou Medical University, Guangzhou 510120, China; yinghuang@gzhmu.edu.cn; 3 Department of Clinical Nutrition, Haikou Affiliated Hospital of Central South University Xiangya School of Medicine, No. 43 Renmin Avenue, Haikou 570208, China; hknutrition@163.com; 4 Guangdong Provincial Key Laboratory of Food, Nutrition and Health, School of Public Health, Sun Yat-Sen University, Guangzhou 510080, China

**Keywords:** bisphenol A, cardiovascular health, NHANES, Life’s Essential 8, environmental exposure, cardiovascular disease

## Abstract

**Background/Objectives:** Cardiovascular diseases are the primary cause of global morbidity and mortality, with cardiovascular health (CVH) remaining well below the ideal level and showing minimal improvement in the U.S. population over recent years. Bisphenol A (BPA), a pervasive environmental contaminant, has emerged as a potential contributor to adverse cardiovascular outcomes. This cross-sectional study delves into the impact of BPA exposure on achieving optimal CVH, as assessed by the Life’s Essential 8 metric, among U.S. adults. **Methods:** Analyzing data from 6635 participants in the National Health and Nutrition Examination Survey (NHANES) collected between 2005 and 2016, BPA exposure was quantified through urinary BPA levels, while optimal CVH was defined using the American Heart Association’s Life’s Essential 8 criteria, scoring between 80 and 100. Multivariable logistic regression and propensity score matching were employed to evaluate the association between BPA exposure and CVH. **Results:** This study reveals that individuals in the highest tertile of urinary BPA levels were 27% less likely to attain optimal CVH compared with those in the lowest tertile (OR, 0.73; 95% CI: 0.59–0.92). This negative association persisted across diverse demographics, including age, sex, and race, mirrored in the link between urinary BPA levels and health factor scores. **Conclusions:** The findings underscore the potential benefits of reducing BPA exposure in enhancing the prevalence of optimal CVH and mitigating the burden of cardiovascular disease. Given the widespread use of BPA, ongoing monitoring of BPA’s impact on CVH is essential. Further studies are necessary to elucidate the long-term and causative connections between BPA and CVH. These insights contribute to understanding the complex interplay between environmental factors and CVH outcomes, informing targeted interventions to mitigate cardiovascular disease risk within the population.

## 1. Introduction

Cardiovascular diseases (CVDs) are the primary cause of mortality around the world, making the prevention and management of these conditions a public health imperative [1]. Recognizing modifiable risk factors is critical, as it allows for the development of strategic interventions aimed at enhancing cardiovascular health (CVH) and providing actionable frameworks to promote CVH. In this realm, the American Heart Association (AHA) has been instrumental, providing valuable benchmarks such as the Life’s Simple 7 (LS7) and its recent iteration, the Life’s Essential 8 (LE8), which not only measure but also guide improvements in CVH [2].

The LS7 has been effective in correlating CVH metrics with various health outcomes, including the incidence of both cardiovascular and non-cardiovascular conditions as well as reduced mortality rates [3,4,5,6]. Recognizing the need for a more holistic approach, the AHA introduced the LE8, which builds on the LS7 through integrating additional determinants of health, thus offering a more nuanced and comprehensive assessment of CVH [2]. The LE8 metric, which evaluates CVH through four health behaviors—diet, physical activity, nicotine exposure, and sleep health—and four health factors—body mass index (BMI), non-high-density lipoprotein cholesterol, blood glucose, and blood pressure—has shown strong associations with reduced incidences of chronic diseases and increased life expectancy [7,8,9,10,11]. However, despite the theoretical advantages of the LE8, overall CVH in the U.S. population remains well below ideal level, with minimal improvement over the past decade [12,13]. This highlights the urgent need for new insights and interventions aimed at improving CVH across diverse populations.

Simultaneously, several environmental contaminants have been implicated in adverse cardiovascular outcomes, including phthalates [14], per- and polyfluoroalkyl substances [15,16], and heavy metals [17,18]. Among these, bisphenol A (BPA)—a ubiquitous compound in everyday items from food containers to medical devices—has emerged as a potential threat to CVH [19]. BPA is an organic synthetic compound (C15H16O2) characterized by two phenol rings connected by a methyl bridge, which allows it to mimic or interfere with hormonal functions, particularly estrogen, via binding to estrogen receptors [20]. The endocrine-disrupting properties of BPA are well-documented, with evidence indicating its potential to disrupt hormonal signaling pathways, induce oxidative stress, promote inflammation, cause cellular dysfunction, and inflict DNA damage, leading to adverse effects across multiple organ systems, including the cardiovascular system [21,22,23,24,25]. In addition to its direct effects on CVD, BPA may indirectly influence CVD by contributing to obesity or diabetes [26]. BPA has been implicated in the dysregulation of lipid metabolism [27,28], blood pressure, glucose homeostasis, and energy balance [29], all of which are critical risk factors for CVD and its complications [30]. Emerging research also suggests that BPA may alter neurodevelopment, thereby affecting appetite [31,32], circadian activity [33,34], cognition [35,36], and other behaviors that may be related to CVD.

Despite the substantial body of experimental evidence from animal studies suggesting BPA’s detrimental effects on CVH, the association remains contentious in human epidemiologic studies. Some studies have reported significant associations between BPA exposure and CVD or cardiometabolic outcomes such as obesity [37,38], type 2 diabetes [39,40], metabolic syndrome [37], dyslipidemia [41,42], hypertension [38,43], and CVD mortality [26,44,45], while others have failed to find such associations or have presented conflicting results [37,46,47,48,49,50]. These inconsistencies may arise from variations in study design, population characteristics, and methods of BPA exposure assessment. This inconsistency in the literature highlights the urgent need for further research employing more comprehensive and standardized metrics to clarify BPA’s role in human CVH.

In light of the conflicting evidence regarding the association of BPA with human CVH and the scarcity of studies utilizing a holistic evaluation of CVH, our study aims to fill this critical gap by hypothesizing that higher BPA exposure is associated with lower CVH, as assessed by the comprehensive LE8 metric. This study aims to address a critical gap via examining the association between BPA exposure and CVH, as defined by the LE8, using data from the National Health and Nutrition Examination Survey (NHANES). The validity of the LE8 as an assessment tool for CVH in NHANES has been validated in several studies [51,52,53]. Through a comprehensive analysis of a nationally representative sample of U.S. adults, we seek to provide robust evidence on the potential relationship between BPA and CVH. By doing so, this study aims to inform public health policies that mitigate the adverse effects of BPA and promote CVH, ultimately addressing a key area of uncertainty in current environmental health research.

## 2. Materials and Methods

### 2.1. Study Design and Participants

**Figure 1:**
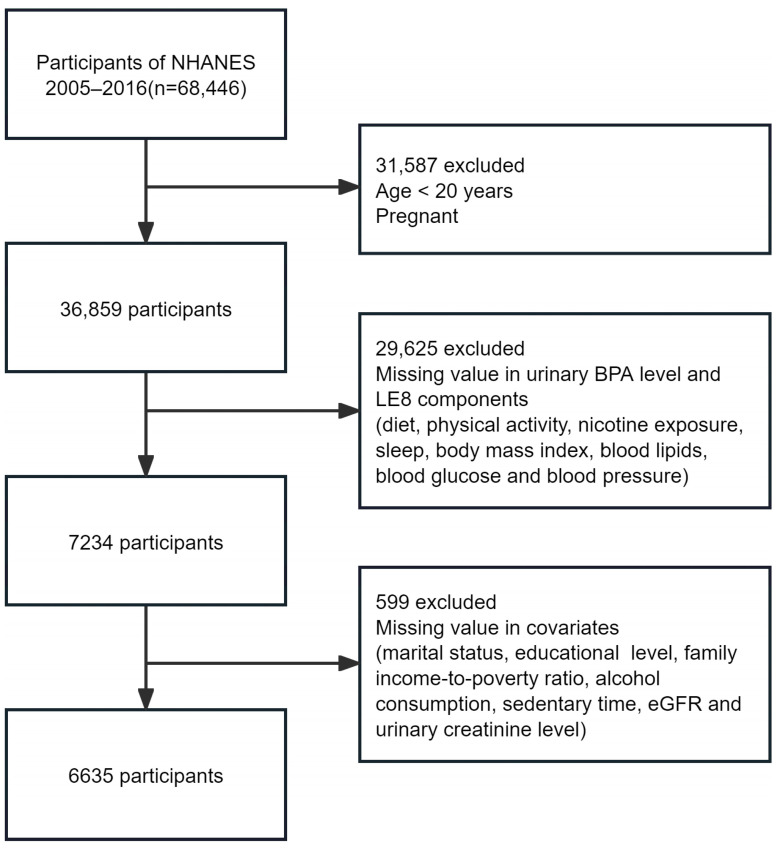
Flow chart of participant selection. Abbreviations: NHANES, National Health and Nutrition Examination Survey; BPA, bisphenol A; LE8, Life’s Essential 8; eGFR, estimated glomerular filtration rate.

Using a multistage probabilistic design, NHANES samples about 5000 U.S. residents annually to represent the non-institutionalized civilian populace. Our study is a cross-sectional, retrospective analysis of data spanning 2005–2016, aggregating six two-year NHANES cycles. These years were selected based on the availability of both urinary BPA measurements and the LE8 metric, enabling the assessment of the relationship between BPA exposure and CVH. Although more recent data exist, they do not include the BPA exposure data necessary for this analysis. From 68,446 participants, we focused on 36,859 non-pregnant adults aged 20 years or above. Following exclusions for missing urinary BPA or CVH data (*n* = 29,625) and incomplete covariate information (*n* = 599), the final sample size was 6635 (Figure 1).

### 2.2. Urinary BPA-Level Measurement

Urinary BPA concentrations were determined via solid-phase extraction, high-performance liquid chromatography, and tandem mass spectrometry, as detailed on the official NHANES website [54]. A single spot urine sample was collected from each participant, processed, and stored at −20 °C until analysis. The analytical method was standardized by the Centers for Disease Control and Prevention, ensuring measurement reliability. Samples below the lower detection limit (0.40 ng/mL for 2005–2012; 0.20 ng/mL for 2013–2016) were assigned a value derived from the square root of the detection limit divided by 2. Approximately 8.3% (552/6635) of the samples were below this threshold. BPA levels were categorized into tertiles for associations analyses with CVH. In linear trend analyses, these tertiles were treated as ordinal variables, with each tertile assigned an integer value (1, 2, or 3).

### 2.3. CVH Assessment (LE8 Score)

In this study, the LE8 metrics were applied to assess CVH. These metrics encompass four health factors and four health behaviors, each evaluated on a scale from 0 to 100. The overall LE8 score was calculated as the arithmetic mean of these eight metrics, providing a comprehensive CVH assessment. The health factors incorporated in the LE8 include BMI, non-high-density lipoprotein cholesterol, blood glucose, and blood pressure. The health behaviors evaluated are diet, physical activity, nicotine exposure, and sleep health. The algorithms for scoring these metrics in the NHANES context have been delineated in previous publications [2,13]. To simplify analysis and result interpretation, the LE8 scores of 80–100 were categorized as “optimal”, while scores below 80 were considered “suboptimal”, based on the AHA’s criteria [2,55]. Dietary quality was appraised using the Healthy Eating Index-2015 (HEI-2015), calculated from 24 h dietary recall data and the food patterns equivalents from the United States Department of Agriculture. The algorithm for the HEI-2015 calculation in NHANES data has been previously published and validated [53,56]. Participants’ physical activity levels, smoking status, sleep patterns, diabetes status, and medication use were self-reported. Weight, height, blood pressure, blood lipids, fasting blood glucose, and hemoglobin A1c were measured in a physical examination setting.

### 2.4. Assessment of Covariates

Baseline data encompassed sociodemographic details, lifestyle, and medical history. Analyzed covariates included age, sex, race/ethnicity, marital and educational level, family income-to-poverty ratio (FITPR), alcohol consumption, sedentary time, urinary creatinine, and estimated glomerular filtration rate (eGFR), based on the prior literature linking these factors to BPA exposure and cardiovascular outcomes [45,57]. Race/ethnicity was divided into four distinct classifications: non-Hispanic white, non-Hispanic black, Mexican-American, and other races. There were three categories for marital status: never married, widowed/divorced/separated, and married or living with a partner. Three categories were used to classify educational attainment: less than high school, high school or equivalent, and college or above. FITPR was calculated as the ratio of family income to the poverty threshold specific to family size and composition as defined by the U.S. Census Bureau. This ratio was stratified into three groups: below 1.3, between 1.3 and 3.5, and above 3.5, representing low, middle, and high socioeconomic status, respectively [58]. Alcohol consumption was quantified according to their reported number of drinks consumed daily or annually, with categories including heavy drinkers (females: ≥1 drink/day, males: ≥2 drinks/day), low-to-moderate drinkers (females: <1 drink/day, males: <2 drinks/day), and non-drinkers (<12 drinks/year). Sedentary time included time spent sitting at desks, in transit, or during leisure activities each day. Creatinine levels were measured using the Jaffe rate reaction, eGFR was calculated using the Modification of Diet in Renal Disease (MDRD) equation, which estimates kidney function based on serum creatinine levels, age, sex, and race [59]. eGFR was expressed in mL/min/1.73 m^2^, with higher values indicating better kidney function.

### 2.5. Statistical Analyses

All statistical analyses accounted for NHANES’ complex sampling design. We used the Rao–Scott chi-square test and linear regression to contrast baseline characteristics for each group, with continuous variables presented as weighted means (standard error [SE]) or weighted medians (interquartile range) and categorical variables presented as weighted percentages (SE). Age-standardized prevalence estimates and corresponding SEs were calculated for BPA exposure categories.

Multivariable logistic regressions were utilized to explore the relationship between urinary BPA levels and optimal CVH, with adjustments for potential confounders. Analyses were stratified by sex, age, and race/ethnicity.

To ensure robustness, we conducted sensitivity analyses, including propensity score matching (PSM), to reduce confounding. PSM was performed using logistic regression to calculate propensity scores, incorporating the aforementioned covariates. Nearest neighbor matching with a caliper width of 0.05 was then applied to minimize differences in propensity scores between matched individuals. A standardized mean difference (SMD) < 0.1 indicates acceptable covariate balance. Additional sensitivity analyses included excluding participants with self-reported CVD (*n* = 704), adjusting for survey cycle, and varying BPA level cut-points.

All statistical tests were two-tailed, with significance set at *p* <  0.05. All analyses were performed with R software version 4.3.2 (the “survey”, “MatchIt”, and “nhanesR” packages). Forest plots were created with Free Statistic software version 1.8.1.

## 3. Results

### 3.1. Baseline Characteristics

Our analysis encompassed 6635 participants, with a balanced sex distribution of 48.94% males and 51.06% females, and a mean age of 47.59 years (SE: 0.34). Within this cohort, 1300 individuals met the criteria for optimal CVH, corresponding to a weighted percentage of 23.38% (SE: 0.01). Notably, participants with optimal CVH tended to be younger, predominantly female, more often non-Hispanic White, married or living with partner, and had higher educational level and FITPR, in conjunction with lower BPA levels (Table 1). Additionally, participants with higher BPA exposure generally had lower scores in overall LE8, health factors, and health behaviors (Table S1). 

### 3.2. Age-Adjusted Prevalence of Optimal CVH

**Figure 2:**
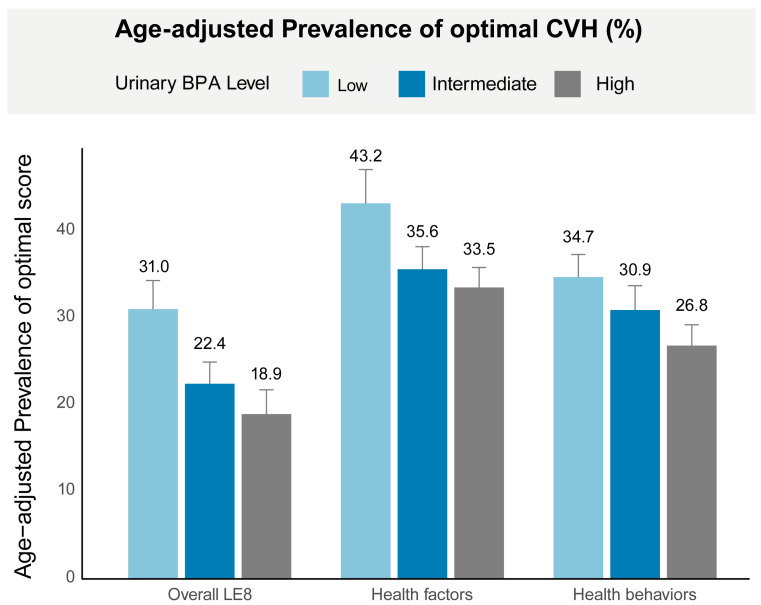
Age-adjusted prevalence of optimal CVH in different urinary BPA levels. Age was adjusted by the direct method to the year 2000 Census population projections using the age groups 20–39, 40–59, and 60+. Numbers at the top of the bars represent the weighted percentage. Bar whiskers represent the 95% confidence level. Abbreviations: LE8, Life’s Essential 8; BPA, bisphenol A.

The age-adjusted prevalence of optimal LE8 score was significantly higher among participants with low urinary BPA levels (31.0%, SE: 1.5) compared to those with intermediate (22.4%, SE: 1.2) and high (18.9%, SE: 1.1) BPA levels. This pattern was consistent across both health factors and health behaviors, with a lower prevalence of optimal scores observed in participants with higher BPA levels (Figure 2).

### 3.3. Association between Urinary BPA Levels and Optimal CVH

**Figure 3:**
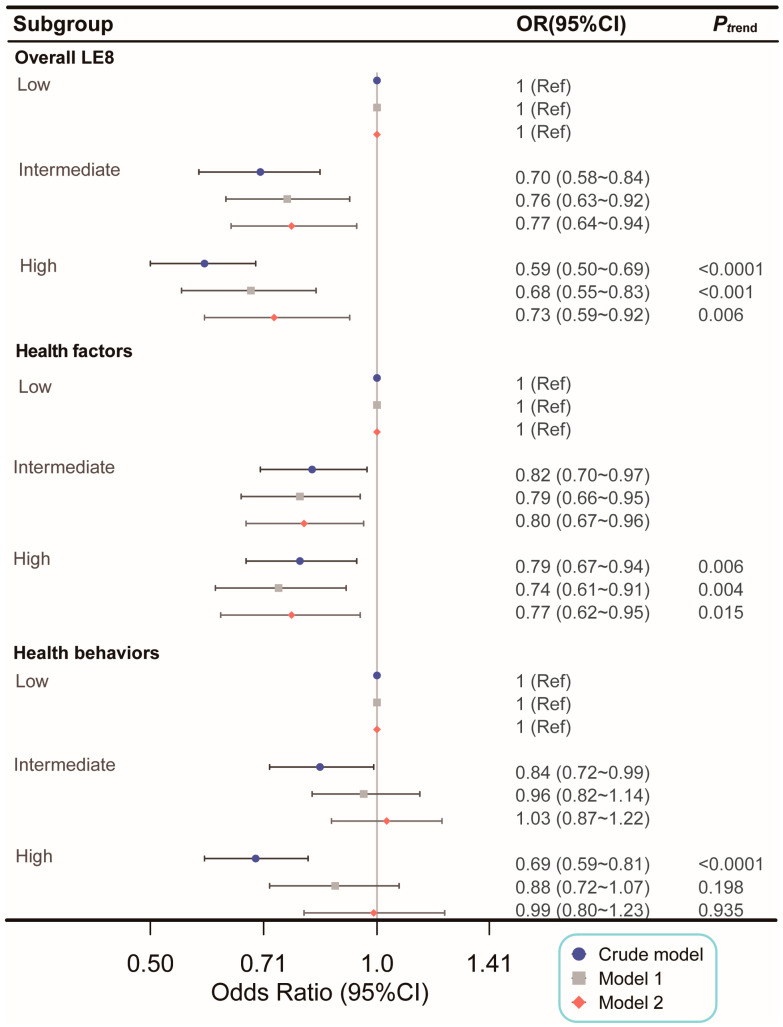
Association of urinary BPA levels with optimal CVH. No covariate was adjusted in crude model. Adjusted covariates in Model 1 included age, sex, race or ethnicity, and urinary creatinine levels. Adjusted covariates in Model 2 encompass those in Model 1, along with marital status, educational level, alcohol consumption, family income-to-poverty ratio, sedentary time, and estimated glomerular filtration rate. The character classification of BPA tertiles was transformed into integers and incorporated into the models to examine the linear trend. Abbreviations: LE8, Life’s Essential 8; OR, odds ratio; CI, confidence interval.

After adjusting for potential confounders, higher urinary BPA levels were significantly associated with lower odds of achieving an optimal LE8 score. Participants in the high BPA group had a 27% reduction in the odds of achieving an optimal LE8 score (OR, 0.73; 95% CI: 0.59–0.92), while those in the intermediate BPA group had a 23% reduction (OR, 0.77; 95% CI: 0.64–0.94) compared to the low BPA group. Similar inverse associations were observed for the health factors component, with a 23% reduction in the odds of achieving optimal health factors in the high BPA group (OR, 0.77; 95% CI: 0.62–0.95). However, the relationship between BPA levels and health behaviors was less definitive. Although a strong inverse association was observed in the crude and minimally adjusted models, this association was no longer significant after full adjustment for covariates (OR, 0.99; 95% CI: 0.80–1.23 for the high BPA group) (Figure 3).

### 3.4. Subgroup and Sensitivity Analysis

**Figure 4:**
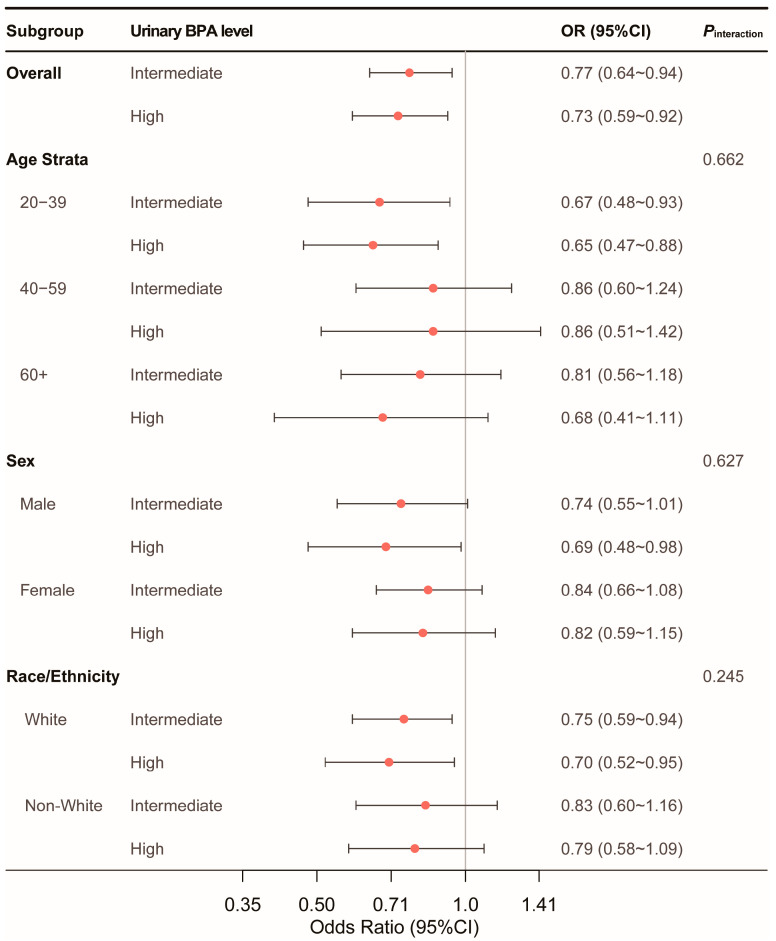
Subgroup analysis of association of urinary BPA levels with optimal CVH. Adjusted covariates included age, sex, race or ethnicity, urinary creatinine levels, marital status, educational level, alcohol consumption, family income-to-poverty ratio, sedentary time, and estimated glomerular filtration rate. Abbreviations: BPA, bisphenol A; OR, odds ratio; CI, confidence interval.

The observed inverse association between urinary BPA levels and optimal CVH persisted across various subgroups, including age, sex, race or ethnicity, with no significant interaction effects detected (Figure 4).

**Figure 5:**
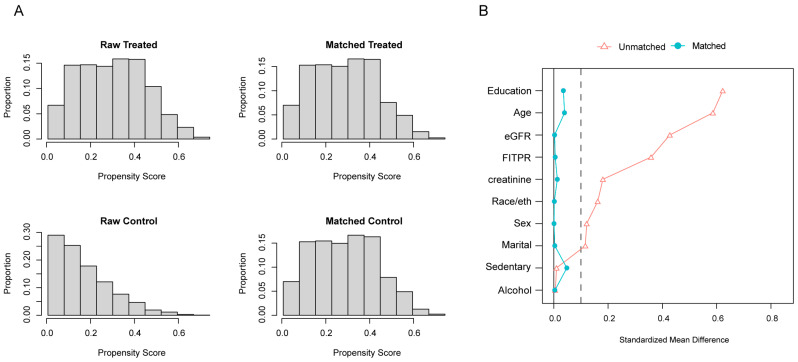
Distribution of propensity score (**A**) and variables standardized mean difference (**B**) before and after matching. Matching for age, sex, race/ethnicity, educational level, marital status, FITPR, alcohol consumption, sedentary time, urinary creatinine levels, and eGFR with caliper = 0.05. Abbreviations: FITPR, family income-to-poverty ratio; eGFR, estimated glomerular filtration rate.

Sensitivity analyses reinforced the robustness of the primary findings. The PSM analysis (*n* = 2490) confirmed significant associations between higher BPA exposure and reduced odds of achieving both optimal overall LE8 and health factors scores, even after controlling for confounders (Table 2). The distribution of propensity scores and standardized mean differences before and after matching are presented in Figure 5, with the matched sample characteristics summarized in Table S2.

Additional sensitivity analyses, including the exclusion of participants with a history of CVD, adjustment for survey cycles, and variations in BPA categorization (quartile-based and log-transformed), consistently indicated that higher BPA exposure is associated with poorer CVH, particularly in terms of overall LE8 and health factors scores. Notably, these associations were not significant for health behaviors, suggesting that BPA’s effects may be more strongly related to biological factors rather than lifestyle behaviors (Table 2).

## 4. Discussion

### 4.1. BPA’s Negative Association with Optimal CVH

In this comprehensive, cross-sectional analysis of a nationally representative U.S. cohort, we discovered a notable inverse relationship between urinary BPA levels and the prevalence of optimal CVH, as defined by the LE8 score, among U.S. adults. This association was consistent across various subgroups, including different age strata, sex, and race or ethnicity, and remained robust after extensive adjustments and sensitivity analyses.

Previous studies have implicated BPA in the development of CVDs, such as myocardial infarction, arrhythmias, dilated cardiomyopathy, atherosclerosis, and hypertension [19]. However, the precise biological mechanisms through which BPA influences CVH remain unclear, though several pathways have been proposed. BPA may disrupt CVH by interacting with estrogen receptors, thereby altering molecular pathways critical for cardiovascular function, leading to vascular dysfunction, inflammation, and oxidative stress [19,22,23,60]. Despite these findings, studies specifically assessing the association between BPA and overall CVH in humans are limited. For instance, Brandi et al. demonstrated a positive correlation between BPA levels and cardiovascular risk in 299 elderly Italians, using two cardiovascular risk scores [61]. Conversely, Olsén et al., using the Framingham Risk Score, found no association between BPA and cardiovascular risk in 1016 elderly Swedes [62]. These discrepancies may be attributed to variations in study design, CVH indicators, population characteristics, and sample size limitations.

To our knowledge, this is the first study to employ the LE8 metric—a comprehensive, multifactorial tool for assessing CVH—to evaluate the relationship between BPA exposure and overall CVH in a large, diverse U.S. adult population. In focusing on the LE8 metric, our research adds novel insights to the existing literature, which has predominantly examined BPA’s association with individual cardiovascular risk factors or specific CVDs. Our findings highlight the relevance of using a more integrative and holistic measure, such as the LE8, in epidemiological studies investigating environmental exposures and CVH. However, due to the cross-sectional nature of the study, we emphasize that these findings reflect associations, not causality.

### 4.2. BPA’s Association with Health Factor and Behavior Scores

This study further distinguished between the effects of BPA exposure on health factor scores and health behavior scores. We observed that while BPA exposure was inversely associated with optimal health factor scores, this relationship was not significant for health behavior scores after full adjustment for covariates. This suggests that BPA’s association with CVH is likely driven more by its direct influence on physiological health factors than on behaviors related to diet, physical activity, smoking, or sleep.

Previous animal studies have demonstrated that BPA exposure can lead to metabolic disturbances, including obesity [63,64], hypertension [65,66], dyslipidemia [67], and diabetes mellitus [68], which are all key components of CVH as defined by the LE8 metric. Recently, Costa et al. demonstrated that BPA exposure negatively affected cardiometabolic outcomes in Portuguese adolescents over time [48]. In this present study, the first to conduct a comprehensive analysis of a large population using the LE8 composite indicator rather than individual risk factors, we observed a persistent negative association between BPA exposure and health factors scores, reinforcing the notion that BPA exposure adversely influences CVH primarily through physiological mechanisms.

Although significant differences in baseline characteristics, particularly diet quality, sleep health, and BMI, suggest BPA’s potential role in disrupting key behaviors or health factors related to CVH (Table S1), the influence of confounding factors and reverse causality cannot be excluded. Notably, while an inverse association between BPA and health behavior scores was observed in crude and minimally adjusted models, this association lost significance after full covariates adjustment, suggesting that other unaccounted factors may confound the relationship. BPA’s potential effects on diet and sleep health may involve neuroendocrine pathways, as Nesan et al. reported that BPA could alter neurodevelopment and affect circadian activity [36]. This is further supported by NHANES data linking BPA exposure to sleep insufficiency [33,34,69]. Additionally, Desai et al. found that BPA exposure increased appetite peptides and decreased satiety peptide expression, suggesting that BPA may indirectly influence health behaviors through neuroendocrine disruption [31]. However, our findings suggest that while BPA may play a role in influencing neuroendocrine function and behavior, its association with CVH is more likely mediated through direct effects on health factors rather than behaviors themselves. This distinction is important for understanding how BPA is related to CVH and for designing targeted public health interventions.

### 4.3. Strengths and Limitations

This study’s strengths lie in its large and diverse sample size and the use of the LE8 metric, which provides a comprehensive assessment of CVH. Importantly, this is the first study to examine the relationship between BPA levels and optimal CVH using the LE8 metric, distinguishing between health factors and health behaviors. By making these distinctions, our study contributes valuable insights into the potential mechanisms through which BPA may influence CVH.

We also accounted for a broad range of covariates, including demographic, socioeconomic, and lifestyle factors, thereby enhancing the precision of our findings regarding BPA’s association with CVH. Additionally, multiple sensitivity analyses, including propensity score matching, were performed to minimize confounding and bolster the robustness of our results.

However, several limitations should be acknowledged. The cross-sectional design limits the ability to infer causal relationships. Additionally, reliance on single-spot urinary BPA measurements may not fully capture temporal variations, and self-reported health behaviors could introduce reporting bias. Despite these limitations, our findings underscore the potential public health benefits of reducing BPA exposure to improve CVH.

Given the pervasive presence of BPA in consumer products, continuous monitoring of its association with CVH is essential. Future longitudinal studies are necessary to establish a causal relationship between BPA exposure and CVH. Moreover, research on community-wide interventions and policy measures to reduce BPA exposure could provide valuable strategies for promoting CVH at the population level.

## 5. Conclusions

Urinary BPA levels were independently and negatively associated with both the optimal overall LE8 scores and the optimal health factor scores in U.S. adults in this study. Efforts to lower BPA exposure have potentially beneficial effects on increasing the prevalence of optimal CVH and reducing the burden of CVDs. Continuous monitoring of the impact of BPA on CVH is warranted given the persistent presence of BPA in daily life. Further research is needed on the longitudinal and causal associations between BPA and optimal CVH.

## Figures and Tables

**Table 1:** Baseline characteristics of the study participants according to cardiovascular health metrics ^a^.

Characteristics	Overall (*n* = 6635)	Life’s Essential 8 Score ^b^		*p* Value
		Optimal (*n* = 1300)	Suboptimal (*n* = 5335)	
Mean (SE)	68.44 (0.30)	86.81 (0.18)	62.84 (0.22)	<0.0001
Age (years)	47.59 (0.34)	42.04 (0.61)	49.28 (0.33)	<0.0001
Sex (%)				<0.0001
Male	48.94 (0.02)	39.93 (1.59)	51.68 (0.88)	
Female	51.06 (0.02)	60.07 (1.59)	48.32 (0.88)	
Race/ethnicity (%)				<0.0001
Non-Hispanic White	71.90 (0.03)	75.15 (1.55)	70.90 (1.56)	
Non-Hispanic Black	9.98 (0.01)	5.66 (0.60)	11.30 (0.88)	
Mexican American	7.43 (0.01)	6.26 (0.76)	7.78 (0.73)	
Other	10.69 (0.01)	12.92 (1.07)	10.01 (0.69)	
Educational level (%)				<0.0001
Less than high school	14.47 (0.01)	6.35 (0.74)	16.94 (0.85)	
High school or equivalent	22.87 (0.01)	10.93 (1.08)	26.52 (1.02)	
College or above	62.66 (0.02)	82.72 (1.34)	56.54 (1.41)	
Marital status (%)				<0.0001
Married/living with partner	66.08 (0.03)	67.12 (1.78)	65.76 (1.07)	
Widowed/divorce/separated	17.67 (0.01)	9.75 (0.92)	20.08 (0.63)	
Never married	16.25 (0.01)	23.14 (1.68)	14.15 (0.84)	
FITPR, (%)				<0.0001
<1.3	17.96 (0.01)	11.93 (1.21)	19.80 (0.93)	
1.3–3.5	36.30 (0.01)	29.23 (1.60)	38.46 (1.01)	
>3.5	45.74 (0.02)	58.84 (1.86)	41.74 (1.30)	
Alcohol consumption (%)				0.03
Non-drinker	21.32 (0.01)	18.68 (1.40)	22.13 (0.81)	
Low-to-moderate drinker	68.51 (0.02)	72.22 (1.49)	67.38 (0.95)	
Heavy drinker	10.16 (0.01)	9.10 (1.00)	10.48 (0.61)	
Sedentary time, minutes/day	341.29 (4.49)	342.23 (7.40)	341.00 (4.84)	0.87
Urinary creatinine level (mg/dL)	120.35 (1.47)	106.24 (2.77)	124.65 (1.62)	<0.0001
eGFR (mL/min/1.73 m^2^)	93.89 (0.42)	98.54 (0.71)	92.47 (0.43)	<0.0001
Urinary BPA level (ng/mL)	1.50 (0.70, 3.00)	1.20 (0.60, 2.50)	1.60 (0.80, 3.20)	<0.0001

Abbreviations: FITPR, family income-to-poverty ratio; eGFR, estimated glomerular filtration rate; BPA, bisphenol A. ^a^ Continuous variables were presented as weighted mean (standard error) and categorical variables were presented as weighted percentage (standard error). ^b^ Range of Life’s Essential 8 score: Optimal, 80–100; Suboptimal, 0–80.

**Table 2:** Sensitivity analysis of urinary BPA levels with cardiovascular health.

	OR (95% CI)		
	Optimal LE8	Optimal Health Factors	Optimal Health Behaviors
Propensity score matching ^a^			
Low	1.00 (Reference)	1.00 (Reference)	1.00 (Reference)
Intermediate	0.77 (0.64, 0.94)	0.80 (0.67, 0.96)	1.03 (0.87, 1.22)
High	0.73 (0.59, 0.92)	0.77 (0.62, 0.95)	0.99 (0.80, 1.23)
Excluding participants with CVD history ^b^			
Low	1.00 (Reference)	1.00 (Reference)	1.00 (Reference)
Intermediate	0.76 (0.62, 0.93)	0.79 (0.65, 0.96)	0.99 (0.83, 1.19)
High	0.71 (0.56, 0.90)	0.77 (0.61, 0.97)	0.98 (0.79, 1.21)
Including the survey cycle ^b^			
Low	1.00 (Reference)	1.00 (Reference)	1.00 (Reference)
Intermediate	0.82 (0.68, 1.00)	0.80 (0.67, 0.97)	1.09 (0.91, 1.31)
High	0.79 (0.63, 1.00)	0.77 (0.62, 0.95)	1.06 (0.85, 1.32)
BPA level categorized by quartiles ^b^			
Quartile 1	1.00 (Reference)	1.00 (Reference)	1.00 (Reference)
Quartile 2	0.80 (0.65, 0.98)	0.77 (0.64, 0.94)	1.02 (0.83, 1.26)
Quartile 3	0.74 (0.58, 0.94)	0.69 (0.55, 0.87)	1.05 (0.85, 1.28)
Quartile 4	0.69 (0.54, 0.87)	0.73 (0.58, 0.91)	0.95 (0.75, 1.20)
Log-transformed BPA ^b^	0.89 (0.82, 0.95)	0.91 (0.85, 0.98)	1.00 (0.93, 1.09)

Abbreviations: BPA, bisphenol A; LE8, Life’s Essential 8; OR, odds ratio; CI, confidence interval. ^a^ Matching for age, sex, race/ethnicity, educational level, marital status, family income-to-poverty ratio, alcohol consumption, sedentary time, urinary creatinine levels, and estimated glomerular filtration rate. ^b^ Covariates adjusted: age, sex, race/ethnicity, educational level, marital status, family income-to-poverty ratio, alcohol consumption, sedentary time, urinary creatinine levels, and estimated glomerular filtration rate.

## Data Availability

All NHANES data for this study are publicly available and can be accessed here: https://wwwn.cdc.gov/nchs/nhanes (accessed on 12 December 2023).

## Supplementary Materials

The following supporting information can be downloaded at: https://www.mdpi.com/article/10.3390/nu16193253/s1, Table S1. Cardiovascular health score of the study participants according to urinary BPA tertiles; Table S2. Characteristics of the matched study population.

## Abbreviations

NHANES: National Health and Nutrition Examination Survey; BPA: bisphenol A; CVD: cardiovascular disease; CVH: cardiovascular health; AHA: American Heart Association; LE8: Life’s Essential 8; LS7: Life’s Simple 7; HEI: Healthy Eating Index; MET: metabolic equivalent; BMI: body mass index; FITPR, family income-to-poverty ratio; eGFR: estimated glomerular filtration rate; SE: standard error; OR: odds ratio; CI: confidence interval.
