# Supplementary material for: The Adverse Impact of Bisphenol A Exposure on Optimal Cardiovascular Health as Measured by Life’s Essential 8 in U.S. Adults: Evidence from NHANES 2005 to 2016
Source: Nutrients. 2024 Sep 26;16(19):3253. doi: 10.3390/nu16193253 (PMC11478777; doi:10.3390/nu16193253)
Supplement: Supplementary file 1 [file nutrients-16-03253-s001.zip › nutrients-3168187-supplementary.pdf]

Supplementary Information

Table S1. Cardiovascular health score of the study participants according to urinary BPA tertiles [a].

Metrics	Overall (*n* = 6635)	Urinary BPA Level [b]			*p* Value
		Low (*n* = 2329)	Intermediate (*n* = 2184)	High (*n* = 2122)	
Overall LE8	68.44 (0.30)	70.06 (0.46)	67.87 (0.43)	67.13 (0.38)	<0.0001
Health factors	70.52 (0.33)	71.73 (0.55)	69.66 (0.57)	70.01 (0.53)	0.01
Health behaviors	66.36 (0.45)	68.38 (0.60)	66.08 (0.64)	64.25 (0.62)	<0.0001
HEI-2015 diet	39.17 (0.66)	42.73 (0.91)	39.14 (0.96)	34.94 (0.91)	<0.0001
Physical activity	72.03 (0.67)	73.09 (1.11)	71.19 (1.09)	71.69 (1.14)	0.37
Nicotine exposure	70.93 (0.78)	72.43 (1.23)	71.04 (1.02)	69.00 (1.08)	0.06
Sleep health	83.31 (0.42)	85.26 (0.62)	82.93 (0.69)	81.39 (0.62)	<0.001
Body mass index	61.22 (0.58)	66.17 (0.97)	58.76 (1.00)	57.95 (1.05)	<0.0001
Blood lipids	64.41 (0.50)	65.07 (0.88)	63.89 (0.85)	64.19 (0.86)	0.59
Blood glucose	86.29 (0.37)	86.64 (0.59)	86.01 (0.66)	86.16 (0.62)	0.72
Blood pressure	70.17 (0.53)	69.04 (0.83)	69.97 (0.92)	71.74 (0.85)	0.07

Abbreviations: BPA, bisphenol A; LE8, Life's Essential 8; HEI-2015, Healthy Eating Index-2015.

[a] Variables were presented as weighted mean (standard error) for normal distribution variables.

[b] Range of urinary BPA level: Low, <1.0 ng/mL; Intermediate, 1.0-2.5 ng/mL; High, >2.5 ng/mL.

Table S2. Characteristics of the matched study population [a].

Characteristics	Life's Essential 8 Score [b]		SMD
	Optimal (n = 1243)	Suboptimal (n = 1243)	
Age (years)	42.56 (0.61)	42.24 (0.49)	0.037
Sex (%)			0.02
Male	41.18 (1.65)	39.11 (1.72)	
Female	58.82 (1.65)	60.89 (1.72)	
Race/ethnicity (%)			0.001
Non-Hispanic White	75.61 (1.63)	70.19 (2.06)	
Non-Hispanic Black	5.81 (0.63)	8.42 (0.88)	
Mexican American	6.13 (0.76)	8.46 (1.07)	
Other	12.45 (1.11)	12.92 (1.19)	
Educational level (%)			0.01
Less than high school	6.61 (0.77)	5.66 (0.69)	
High school or equivalent	11.38 (1.12)	16.39 (1.42)	
College or above	82.02 (1.40)	77.95 (1.61)	
Marital status (%)			0.017
Married/living with partner	67.66 (1.76)	64.25 (1.76)	
Widowed/divorce/separated	10.11 (0.96)	14.15 (1.11)	
Never married	22.23 (1.63)	21.60 (1.52)	
Family income-to-poverty ratio (%)			0.018
<1.3	12.32 (1.27)	10.67 (0.94)	
1.3-3.5	29.50 (1.68)	33.37 (1.89)	
>3.5	58.18 (1.95)	55.96 (2.02)	
Alcohol consumption (%)			<0.001
Non-drinker	18.49 (1.44)	20.41 (1.37)	
Low to moderate drinker	72.24 (1.51)	69.93 (1.66)	
Heavy drinker	9.27 (1.04)	9.66 (1.30)	
Sedentary time, minutes/day	339.81 (7.26)	333.14 (7.94)	0.015
Urinary creatinine levels (mg/dL)	107.96 (2.86)	106.06 (2.04)	0.047
eGFR (mL/min/1.73 m2)	98.19 (0.70)	98.82 (0.72)	0.025

Abbreviations: FITPR, family income-to-poverty ratio; eGFR, estimated glomerular filtration rate; BPA, bisphenol A.

[a] Continuous variables were presented as weighted mean (standard error), and categorical variables were presented as weighted percentage (standard error).

[b] Range of Life's Essential 8 score: Optimal, 80-100; Suboptimal, 0-80.
